# Viviparity in the dermapteran *Arixenia esau*: respiration inside mother’s body requires both maternal and larval contribution

**DOI:** 10.1007/s00709-019-01402-1

**Published:** 2019-06-19

**Authors:** Mariusz K. Jaglarz, Waclaw Tworzydlo, Agnieszka Rak, Malgorzata Kotula-Balak, Malgorzata Sekula, Szczepan M. Bilinski

**Affiliations:** 1 Department of Developmental Biology and Invertebrate Morphology, Institute of Zoology and Biomedical Research, Faculty of Biology, Jagiellonian University, Gronostajowa 9, 30-387 Krakow, Poland; 2 Department of Physiology and Toxicology of Reproduction, Institute of Zoology and Biomedical Research, Faculty of Biology, Jagiellonian University, Gronostajowa 9, 30-387 Krakow, Poland; 3 Department of Endocrinology, Institute of Zoology and Biomedical Research, Faculty of Biology, Jagiellonian University, Gronostajowa 9, 30-387 Krakow, Poland

**Keywords:** Respiration, Dermaptera, Tracheae, Hemocyanin, Fat body, Insects

## Abstract

Earwigs (Dermaptera) use different strategies to increase their reproductive success. Most species lay eggs; however, viviparity of the matrotrophic type has been reported in two groups: Hemimeridae and Arixeniidae. In Arixeniidae, offspring develop in two separate places: inside an ovary (the intraovarian phase) and within a uterus (the intrauterine phase). Both morphological and physiological aspects of viviparity in Arixeniidae have begun to be unraveled only recently. Here, we characterize how the first instar larvae of *Arixenia esau*, developing inside the mother’s reproductive system, manage respiration and gas exchange. Using modern light and electron microscopy techniques as well as immunological approach, we provide a detailed account of the maternal and larval tissue interactions during the intrauterine development. We demonstrate that respiration in the *Arixenia* first instar larvae relies on the extensive tracheal system of the mother as well as a respiratory pigment (hemocyanin) present within the body cavity of the larvae. Our results indicate that the larval fat body tissue is the likely place of the hemocyanin synthesis. Our study shows that characteristic cone-shaped lobes of the outgrowths located on the larval abdomen are a part of a placenta-like organ and mediate the gas exchange between the maternal and larval organisms. Based on the obtained results, we propose that *Arixenia esau* evolved a unique biphasic system supporting respiration of the first instar larvae during their development inside the mother’s reproductive tract.

## Introduction

Insects evolved several strategies to maximize their reproductive success. Most insect species are oviparous. They lay eggs filled with reserve materials and allow embryos to develop, usually unattended, in the external environment. In several insect groups, however, females retain progeny inside their body and support their development with various nutrients for an extended period of time (reviewed in Retnakaran and Percy 1985). This mode of reproduction, termed the matrotrophy or matrotrophic viviparity, has many advantages over oviparity, e.g., continuous protection of progeny inside a mother’s body and maintenance of the most favorable conditions for development. However, viviparity creates many physiological challenges associated with sustaining adequate nutrition for the developing embryos, metabolic waste removal, and proper gas exchange (for review, see Wheeler 2003; Ostrovsky 2013; Blackburn 1999, 2015; Ostrovsky et al. 2016). In insects, both morphological and physiological modifications related to matrotrophic viviparity are poorly characterized.

In recent years, we have been intensively studying different aspects of matrotrophy in earwigs (Dermaptera)—hemimetabolous insects with a varied pattern of reproduction. Most dermapterans lay eggs, but in two taxa, the Hemimeridae and Arixeniidae, females give birth to living larvae. These viviparous dermapterans live non-parasitically on the body surface of giant murid rats (hemimerids) or certain bats (arixeniids). It has been suggested that viviparous reproduction and matrotrophy evolved in these dermapterans as an adaptation to their epizoic mode of life (Nakata and Maa 1974; Richards and Davies 1977). In previous studies, we have analyzed morphology of the reproductive system, embryogenesis, and the relations between maternal and embryonic tissues in the hemimerid, *Hemimerus talpoides* (Bilinski et al. 2017, 2018). We showed that in this species, the oocytes are completely devoid of yolk spheres and lipid droplets as well as continuous egg envelopes. Mature oocytes are instead surrounded by a highly modified follicular epithelium which participates in nourishment of the early embryo (Bilinski et al. 2017). Interestingly, the complex embryonic development of *H. talpoides* occurs within the ovary, in the terminal ovarian follicle, and is dependent on transfer of nutrients from maternal tissues (for further details, see Hagan 1951; Bilinski et al. 2017, 2018).

Recent morphological analyses of the reproductive system in *Arixenia esau*, a representative of the Arixeniidae, revealed a completely different pattern of embryonic development from that described in *Hemimerus*. First of all, only the very early embryos develop in the ovarian tubes (ovarioles) of the ovary, within terminal ovarian follicles (for further details, see Tworzydlo et al. 2013a, 2013b). The best part of the embryo and first instar larva development takes place in transformed lateral oviducts, collectively termed the uterus (Tworzydlo et al. 2013a, 2013b; Tworzydlo 2015). *Arixenia* embryonic development was therefore separated into two consecutive phases: intraovarian and intrauterine (Tworzydlo et al. 2013a, 2013b; Bilinski and Tworzydlo 2019). During the intraovarian phase, the embryos rely on reserve materials accumulated during oogenesis in the oocyte cytoplasm. However, the progeny receives nutrients directly from the mother’s body while in the uterus (Bilinski and Tworzydlo 2019).

The characteristic feature of the *Arixenia* advanced embryos and first instar larvae is the presence of characteristic outgrowths on the dorsal side of the first eight abdominal segments (Bilinski and Tworzydlo 2019). The outgrowths are ramified into four morphologically distinct lobes which, in larvae, protrude from the abdominal surface. As larvae grow, the outgrowth lobes adhere to the uterine epithelium, forming distinct contact points between maternal and larval tissues. It was suggested that these contact points collectively constitute a dispersed placental analogue and at least some of the lobes may be engaged in the nourishment of the *Arixenia* offspring (Bilinski and Tworzydlo 2019).

The physiological aspects of the viviparous matrotrophy in Arixeniidae remain largely unexplored. Previously, we have demonstrated that in *A. esau*, the excretion of the first instar larvae, developing in the uterus, is independent of the maternal excretory system and involves both larval Malpighian tubules and specialized cells at the midgut-hindgut junction (for further details, see Jaglarz et al. 2018).

Another vital aspect of development is respiration. Gas exchange is indispensable for all animal organisms with aerobic metabolism. It involves the entry of oxygen into the body, its delivery to all cells engaged in oxidative metabolism and the elimination of carbon dioxide. In the majority of animals, due to their complex, multilayer cellular organization, the simple diffusion of respiratory gases across the surface of the body is insufficient to support all physiological processes requiring oxygen. Therefore, in most species, the respiratory requirements are met by an elaborate blood system containing respiratory pigments, such as hemoglobins or hemocyanins, and providing oxygen to all cells. In insects, a completely different respiratory system, termed the tracheal system, evolved. It opens on the surface of the body through spiracular apertures and relies on branching tubules of increasingly smaller diameter and thinner walls, which bring air/oxygen directly to all tissues and cells (reviewed in Harrison 2003). The walls of the tubes are reinforced by cuticular taenidia which resist collapsing and thus allowing free passage of air. The tracheal system, in principle, does not require a presence of respiratory pigments. However, in recent years, quite unexpectedly, several reports indicated the presence of respiratory proteins, including hemocyanins, also in several insect groups (for review, see Burmester 2002; Pick et al. 2009). It appears therefore that, at least in some insects, the physiology of oxygen supply might be more complex than previously expected.

In the context of respiration, viviparity poses a real challenge: embryos develop deep inside parental body, separated from the direct contact with air and usually surrounded tightly by mother’s tissues. The problem of ensuring adequate oxygen levels for progeny was solved in some animals, e.g., certain mammals, by the formation of a placenta, a complex temporal organ, which mediates, among others, gas exchange via mother’s blood system (Gilbert 2014). The aim of this study was to gain insight into the structural modifications and physiological processes accompanying respiration and gas exchange in the *Arixenia* first instar larvae as they develop inside the mother’s reproductive system. Because the intraovarian development was characterized in detail previously (Tworzydlo et al. 2013a, 2013b), here, we focus on the intrauterine phase.

## Material and methods

### Animals

The adult females of *Arixenia esau* Jordan, 1909 were collected from the walls of small caves (inhabited by bat colonies) in Bintulu District area, Sarawak, Malaysia. Five fully grown females and more than 20 first instar larvae were used in our studies. Fragments of dissected uteri and isolated larvae were fixed in appropriate chemicals for further analyses.

### Light and electron microscopy

The dissected material was fixed in a mixture of 2.5% glutaraldehyde and 1.5% formaldehyde in 0.1 M phosphate buffer (pH 7.3). Samples were rinsed in phosphate buffer with sucrose (5.8 g/100 ml) and postfixed in a mixture of 1% osmium tetroxide and 0.8% potassium ferrocyanide for 30 min at 4 °C. After dehydration in the graded series of ethanol and acetone, the material was infiltrated in a freshly prepared mixture of acetone and Epon 812 (Serva, Heidelberg, Germany), placed in a vacuum drier for 6 h (Thermo Fisher Scientific, Waltham, Massachusetts, USA), and embedded in Epon 812. Semithin sections (0.7–1 μm thick) were stained with 1% methylene blue and examined under a Nikon Eclipse Ni (Tokyo, Japan) or a Leica DMR light microscope (LM) (Heidelberg, Germany). Ultrathin sections (80 nm thick) were contrasted with uranyl acetate and lead citrate according to standard protocols and analyzed with a transmission electron microscope (TEM) Jeol JEM 2100 (Tokyo, Japan) at 80 kV.

### Scanning electron microscopy

For the SEM analyses, five larvae and five fragments of isolated uteri were fixed and postfixed as described above. After dehydration, the material was critical-point dried, coated with gold and examined with a Hitachi S-4700 (Tokyo, Japan) scanning electron microscope at 25 kV (see Jaglarz et al. 2018 for further details).

### Immunolocalization of hemocyanin subunits

For the immunohistochemical analyses, the material was fixed in 4% formaldehyde. Samples were dehydrated in series of ethanol and HistoChoice® Clearing Agent (Sigma-Aldrich) and embedded in paraplast. The paraplast blocks were cut into 5-μm-thick sections. Slide-mounted sections were deparaffinized (dewaxed) in HistoChoice® Clearing Agent (Sigma-Aldrich), rehydrated gradually through a series of ethanol dilutions and rinsed in water. Blocking of non-specific binding sites was performed with casein blocking buffer (Thermo Fisher) overnight at 4 °C prior to the incubation with anti-HC1 and HC2 antibodies (the antibodies were raised against the hemocyanin subunits of the Dubia roach, *Blabtica dubia*; a generous gift from Prof. T. Burmester, Hamburg University, Germany) diluted 1:500. In parallel performed control experiments, the primary antibody was omitted. After overnight incubation at 4 °C in a humidified chamber, sections were washed with PBS with 0.1% Triton X-100 and 0.05% Tween 20 and incubated with Alexa Fluor 488 or Cy3 goat anti-rabbit secondary antibodies (Life Technologies) for 4 h at room temperature. After rinsing with PBS, the sections were mounted in ProLong Gold antifade reagents with DAPI (Invitrogen) and analyzed in the DMR Leica epifluorescence microscope equipped with appropriate filters. Each staining experiment was performed in triplicate.

### Western blot analysis

To quantify HC1 and HC2 protein expression, first instar larvae dissected from the uteri were homogenized twice in ice-cold lysis buffer which contained 50 mM Tris-HCl (pH 7.5), 100 mM NaCl, 0.5% Na-deoxycholate, 0.5% NP-40, 0.5% SDS, and protease inhibitor (EDTA-free). Lysates were cleared by centrifugation at 15.000×*g* at 4 °C for 30 min, and the protein content was determined by a protein assay using bovine serum albumin (BSA) as a standard (Bradford method). Sixty micrograms of protein from each sample was reconstituted directly in the appropriate amount of sample buffer and separated in Mini-Protean TGX System Precast Protein Gels (Bio-Rad, Hercules, CA, USA) and transferred to Trans-Blot Turbo Mini PVDF Transfer Packs (Bio-Rad, Hercules, CA, USA). The membranes were washed and blocked in 0.02 M Tris-buffered saline containing 5% BSA and 0.1% Tween 20 and then incubated overnight at 4 °C with anti-HC1 or HC2 antibodies diluted 1:500. Next, the membranes were washed with TBST (Tris-buffered saline containing 0.1% Tween 20) and incubated for 1 h with a horseradish peroxidase-conjugated goat anti-rabbit antibody (Santa Cruz, USA) diluted 1:1000. Signals were detected by chemiluminescence using WesternBright Quantum HRP substrate (Advansta Inc., Menlo Park, USA) and visualized using the Chemidoc™ XRS + System (Bio-Rad, Hercules, USA).

### Localization of collagen fibers

For the detection of collagen fibers, we used Trichrome Stain (Masson) Kit (Sigma-Aldrich). Briefly, paraplast sections (see above) were deparaffinized (dewaxed) in HistoChoice® Clearing Agent (Sigma-Aldrich), rehydrated gradually through a series of ethanol dilutions, and rinsed in deionized water. Next, the sections were stained in Weigert’s iron hematoxylin and Briebrich Scarlet Acid Fuchsin for 10 min each. After placing the sections in phosphotungstic-phosphomolybdic acid solution, they were stained in anilin blue and placed in 1% acetic acid for 2 min. The slides were mounted and examined under a Nikon Eclipse Ni (Tokyo, Japan) or a Leica DMR light microscope (LM) (Heidelberg, Germany).

## Results

**Fig. 1:**
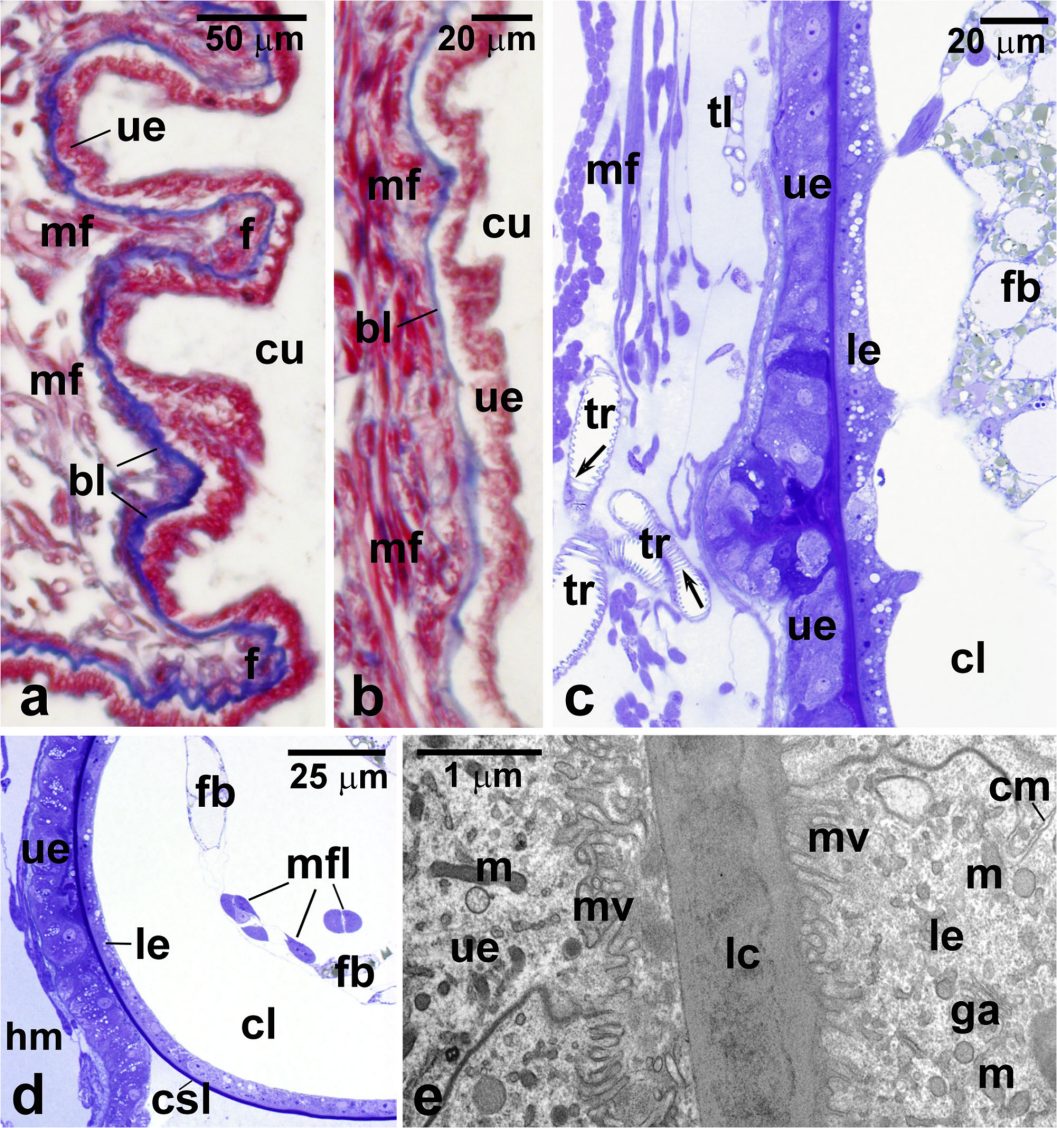
Tissues of the mother-larva interface. **a** The initial stage of intrauterine development: the uterus wall is folded and composed of a prismatic epithelium (ue) supported by a prominent basal lamina (bl, stained blue) covered outside by a thick layer of striated muscle fibers (mf). **b** The advanced stage of intrauterine development: the uterus wall is stretched, with no folds; the uterine epithelium (ue) is supported with a thin basal lamina (bl). **c**, **d** The contact zone between the wall of the uterus (ue) and cone-shaped lobe (csl) of the larval abdominal outgrowth. Numerous tracheal tubules (tr) and tracheoles (tl) are present between muscle fibers (mf) of the uterine wall and reach the basal part of the uterine epithelium (ue). Note that the spacious cavity (cl) of cone-shaped lobe contains fat body strips (fb) and muscle fibers (mfl); only part of the epithelium covering the cone-shaped lobe (le) adheres closely to the uterine epithelium (ue). **e** Electron micrograph showing the contact zone between the maternal and larval tissues. The apical parts of the epithelia lining the uterus (ue) and covering the cone-shaped lobe (le) are equipped with microvilli (mv) and separated only by a thin layer of the larval cuticle (lc). cm, cell membrane; cu, cavity of the uterus; f, fold of the uterus; ga, Golgi apparatus; hm, hemocoel of the mother; le, epithelium covering the cone-shaped lobe; m, mitochondrion; mf, muscle fibers; ue, uterine epithelium; arrows—taenidia. **a**, **b** Paraffin sections; trichrome method, LM; **c**, **d** Epon sections; methylene blue, LM; **e** ultrathin section, TEM

**Fig. 2:**
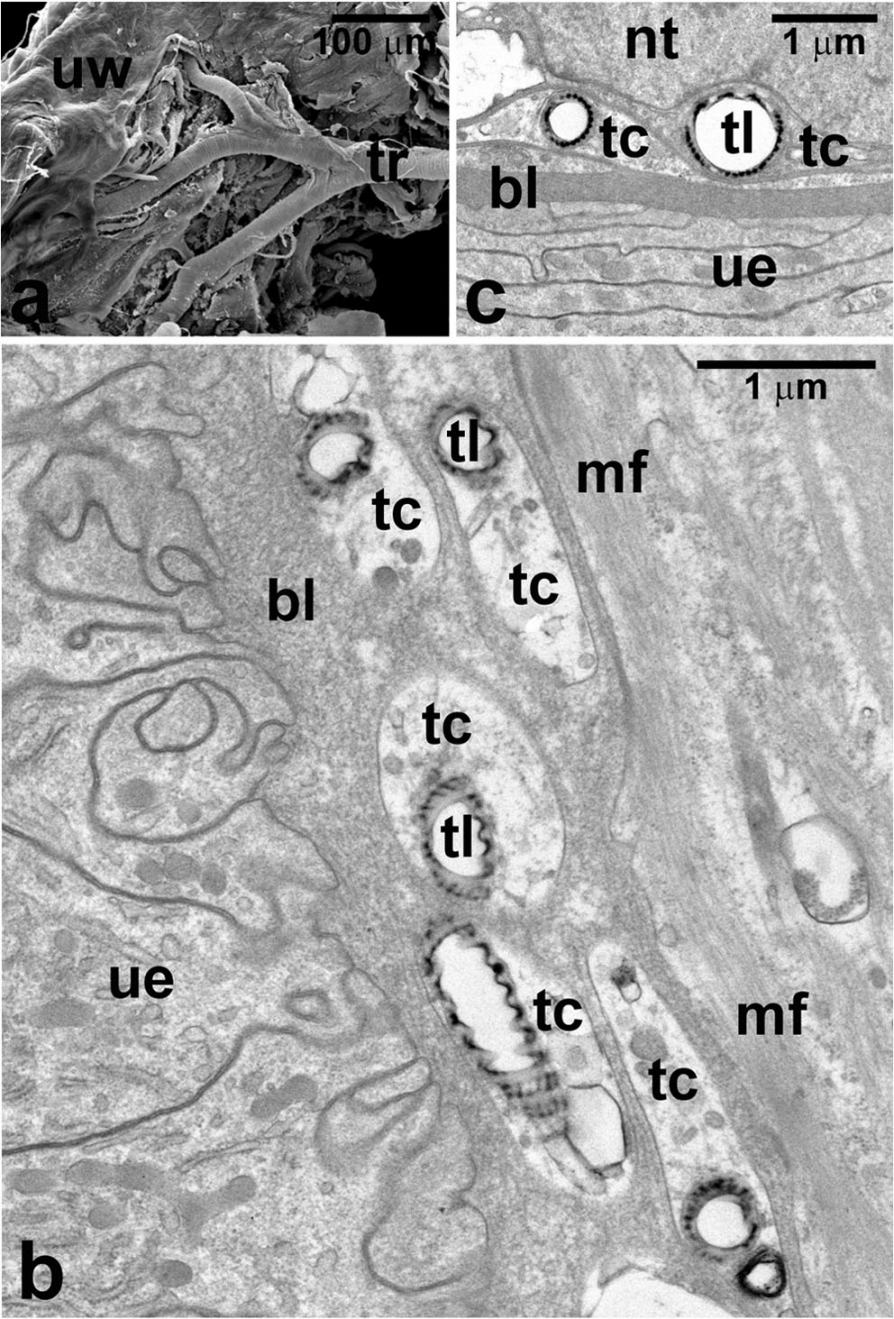
Tracheal elements of the *Arixenia* uterus. **a** A large tracheal trunk (tr) branching and penetrating the uterine wall (uw). **b**, **c** Tracheoles (tl), surrounded by tracheal end cells (tc), adhere to or penetrate the basal lamina (bl) supporting the uterine epithelium (ue). mf, muscle fibers of the uterine wall; nt, nucleus of a tracheal end cell. **a** SEM; **b**, **c** ultrathin sections, TEM

At the initial stage of the *Arixenia* intrauterine development, the uterine wall is highly folded and composed of prismatic epithelium, supported by a prominent basal lamina covered outside by a thick layer of the striated muscle fibers (Fig. 1a). The trichrome staining revealed that the basal lamina (around 6 μm in thickness) contains substantial amount of collagen (Fig. 1a). The *Arixenia* uterus is covered with a rich system of tracheae. The larger trunks of tracheae, 15–20 μm in diameter, reaching the surface of the uterine wall branch repeatedly into increasingly smaller diameter tubes (Figs. 1c and 2a–c). The tracheae insinuate themselves between muscle fibers and end with the finest tracheoles, less than 1 μm in diameter (Figs. 1c and 2a–c). As a result, several layers of tracheal and tracheolar tubes form a dense network penetrating the uterine muscle fibers and the thick basal lamina (Figs. 1c and 2b). The microscopic analysis showed that tracheae are lined with an opaque cuticular layer or intima, reinforced by numerous helical or annular thickenings, i.e., taenidia (Fig. 1c). In contrast, the tracheolar lining is thinner and, in the finest tracheoles, smooth with no taenidial tubercles (Fig. 2b, c). Tracheoles forming the terminal endings of the tracheal system almost approach the uterine epithelium (Figs. 1c and 2b, c). While the distal segments of tracheoles enter and penetrate the basal lamina of the uterine epithelial cells, the proximal ones are surrounded by tracheal end cells (Fig. 2b, c).

After larva hatching (eclosion) from the egg envelope, the multilobed outgrowths, protruding from the dorsal surface of larval abdominal segments, come into contact with the uterine epithelium (Fig. 1c–e). During this stage of the intrauterine development, the larvae grow to a length of up to 15 mm, roughly 1/3 the length of the female abdomen. Such a considerable increase in the larva sizes distends the uterus and straightens its epithelial wall (Fig. 1b). The extended basal lamina is now only 1 μm thick (Fig. 1b).

**Fig. 3:**
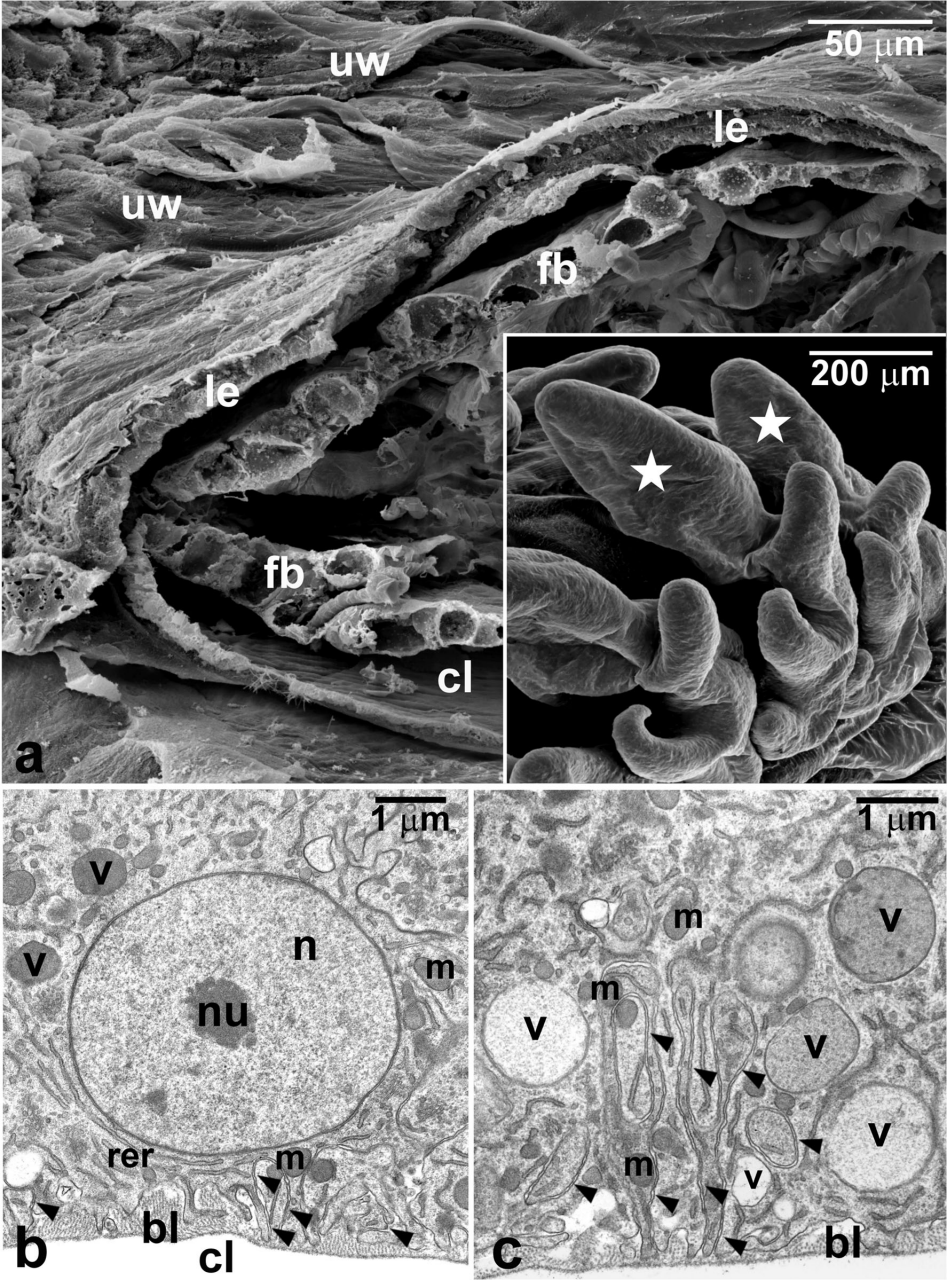
Cone-shaped lobes of the larval abdominal outgrowths. **a** Fractured cone-shaped lobe and the adjacent uterine wall (uw). Inset, part of the larval abdomen; dorsal side of the abdomen is covered with outgrowths, aster—cone-shaped lobe of the abdominal outgrowths. **b**, **c** Basal region of the cone-shaped lobe epithelium. bl, basal lamina; cl, cavity of the cone-shaped lobe; fb, fat body strands; le, epithelium covering the cone-shaped lobe; m, mitochondrion; n, nucleus; nu, nucleolus; rer, cisternae of the rough endoplasmic reticulum; v, vacuole; arrowheads—basal membrane infoldings. **a**, inset SEM. **b**, **c** ultrathin sections, TEM

**Fig. 4:**
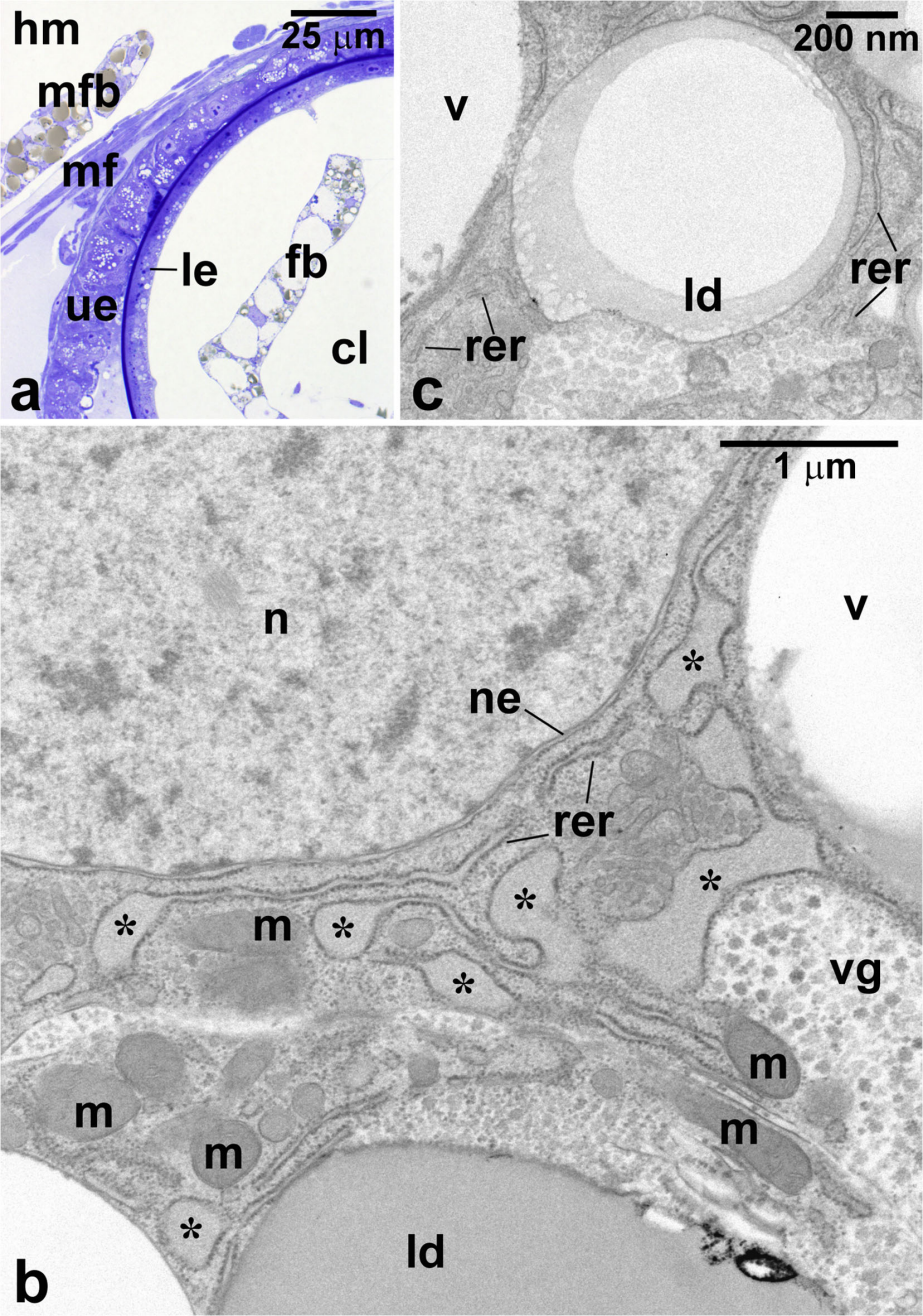
Structure of the fat body tissue in the mother’s body cavity and cavity of the larval cone-shaped lobe. **a** Fragment of the cone-shaped lobe contacting the uterine epithelium (ue) with an adjacent maternal fat body strand (mfb). Note a difference in the gross morphology of the maternal and larval (fb) fat body tissues. **b** Ultrastructure of the cone-shaped lobe adipocyte; note characteristically distended cisternae (asterisks) of the rough endoplasmic reticulum (rer). **c** Fragment of the maternal fat body adipocyte. cl, cavity of the cone-shaped lobe; hm, hemocoel of the mother; ld, lipid droplet; le, epithelium covering the cone-shaped lobe; m, mitochondrion; mf, muscle fibers; n, nucleus; ne, nuclear envelope; rer, cisternae of the rough endoplasmic reticulum; v, vacuole; vg, vacuole containing glycogen aggregates. **a** Semithin section, methylene blue, LM; **b**, **c** ultrathin sections, TEM

Our preliminary analyses have suggested that the largest and cone-shaped lobe (CSL) of the abdominal outgrowths (Fig. 3a inset, asters) might be a good candidate for an organ participating in respiration. Each CSL is covered by a flat one-cell thick epithelium, which surrounds a spacious centrally located cavity filled with hemolymph and connected with the body cavity (the hemocoel) of the first instar larva (Figs. 3a and 4a). Most importantly, each CSL adheres to the uterine wall forming a distinct contact zone (Figs. 3a and 4a). At this zone, the microvilli-equipped apical parts of the epithelial cells of both CSLs and the uterus are directed towards one another and separated only by a relatively thin layer of the larval cuticle, already deposited on the surface of CSLs (Fig. 1e). Thus, the apical region of the CSL epithelial layer is directed outside similarly to an epidermal layer covering the surface of the rest of the body. In contrast, the basal parts of the CSL epithelial cells face the CSL cavity and are supported by a thin basal lamina composed of loosely arranged fine fibers (Fig. 3b, c). A characteristic feature of the basal parts are also numerous membrane infoldings penetrating the cytoplasm (Fig. 3b, c). The cytoplasm comprises a spherical nucleus with a well-developed nucleolus, as well as mitochondria, cisternae of the endoplasmic reticulum (ER), and variably sized vesicles of different electron opacity (Fig. 3b, c). We have not observed any morphological evidence of endocytic activity in the epithelial cells of CSLs.

**Fig. 5:**
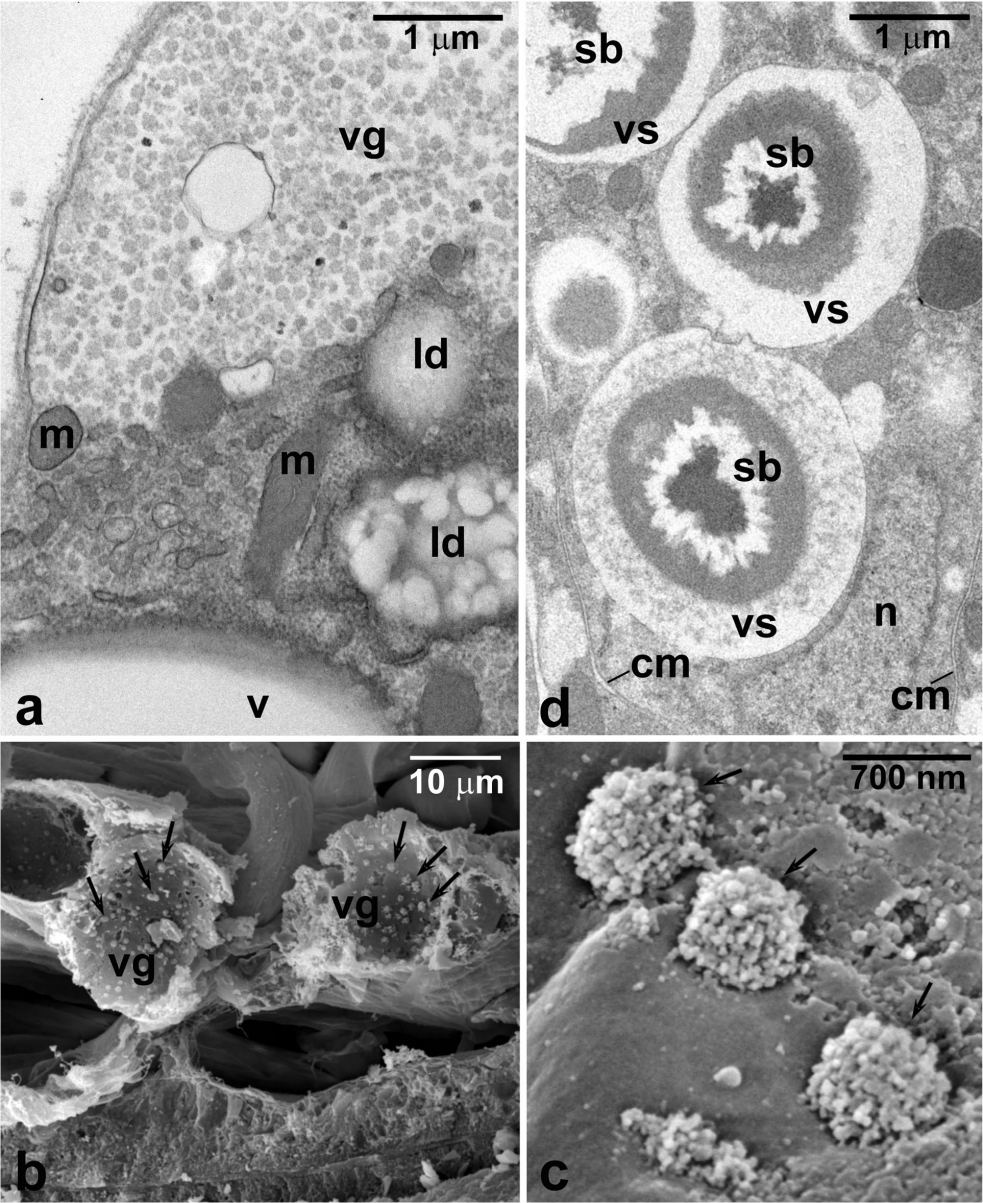
Larval fat body tissue of the cone-shaped lobe. **a** Fragment of the adipocyte with a large vacuole (vg) filled with glycogen aggregates. **b** Fragment of the cone-shaped lobe fat body tissue with fractured adipocyte vacuoles (vg) containing glycogen aggregates (arrows). **c** Higher magnification of the vacuole with broccoli-like rosettes (arrows) of glycogen particles. **d** Fragment of a urocyte filled with vacuoles (vs) containing spherical bodies (sb). cm, cell membrane; ld, lipid droplet; m, mitochondrion; n, nucleus; v, vacuole. **a**, **d** Ultrathin section, TEM; **b**, **c** SEM

Serial section analyses revealed additionally that several stripes of fat body and striated muscle fibers are present within the CSL cavity (Figs. 1c, d and 4a). The larval fat body tissue is composed of two distinct cell types: (1) more abundant polyhedral adipocytes and (2) elongated urocytes, interspersed among adipocytes. The adipocyte cytoplasm is filled with numerous lipid droplets and variably sized vacuoles some of which contain clusters of small particles immersed in an electron-translucent substance (Figs. 4a, b and 5a). The scanning electron microscope analysis of the ruptured adipocyte vacuoles revealed that these clusters take on the characteristic appearance of small cauliflower or broccoli rosettes (Fig. 5b, c). The diameter of the clusters ranges from 600 to 800 nm, while the individual particles have the diameter of 20–30 nm. The fine structure of the clusters as well as results of the measurements suggest that they represent particles of α-glycogen.

The high vacuolization of the adipocytes restricts cytosol to small islands or narrow strands between the vacuoles and lipid droplets. The adipocyte spherical nucleus is surrounded by a regular nuclear envelope, occasionally pierced with nuclear pores (Fig. 4b). The nucleoplasm contains mostly dispersed chromatin with irregularly distributed small clumps of heterochromatin (Fig. 4b). The cytoplasm comprises free ribosomes, mitochondria, and prominent cisternae of ER. The majority of these cisternae are of the rough ER type as evidenced by dense population of ribosomes associated with the external surface of their membranes (Fig. 4b). The distinguishing feature of the adipocyte rough ER cisternae is that a large portion of their lumen is considerably distended and filled with homogenous fine granular material of medium-electron density (Fig. 4b).

Interestingly, the fat body tissue of the mother is morphologically different from that residing in the cavity of CSL. The microscopic analysis of serial semithin sections revealed that maternal adipocytes are less vacuolated but contain more lipid droplets in comparison with the larval fat body adipocytes (Fig. 4a). In addition, the cytoplasm of the maternal adipocytes comprises only sparse cisternae of the rough ER, which have a more regular appearance and are not distended nor filled with fine granular material like the ones inside CSL (Fig. 4c).

In the larval fat body, the cytoplasm of the urocytes is packed with vacuoles containing large spherules (spherical bodies) consisting of material of different electron density and in a various degree of condensation or crystallization (Fig. 5d). The cell nucleus is crescent-shaped and shifted towards one pole of the cell, seemingly by the enlarging vacuoles (Fig. 5d). In contrast to adipocytes, the cytoplasm of urocytes contains few mitochondria and lacks prominent cisternae of ER.

**Fig. 6:**
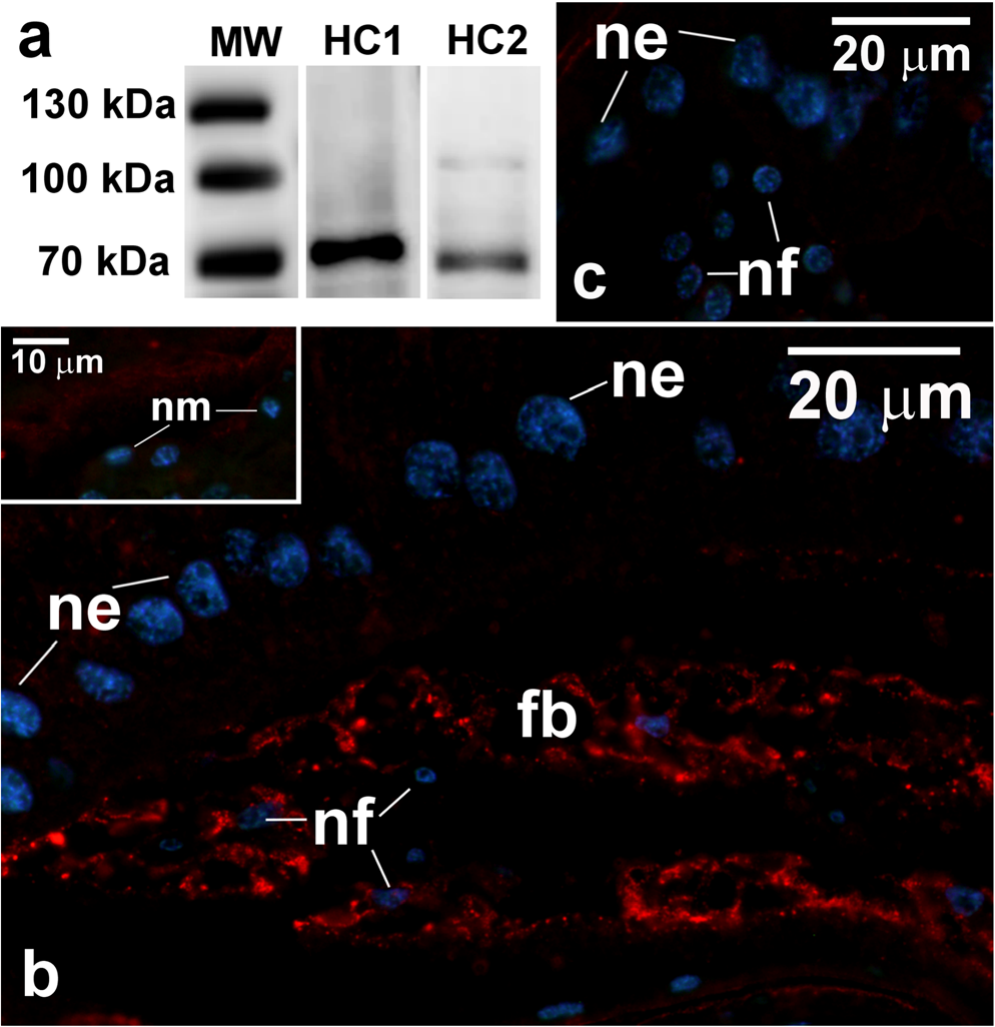
Detection of hemocyanin subunits in *Arixenia* tissues. **a** Immunoblots with antibodies against hemocyanin subunits HC1 and HC2; MW—molecular weight markers. **b** Fragment of the *Arixenia* larval cone-shaped lobe and fat body tissue of the mother (inset) stained with the antibody against HC1 subunit (the secondary antibody conjugated to Cy3) and DAPI to reveal cell nuclei. Note that HC1 was not detected in the fat body tissue of the mother (inset). **c** Negative control staining: fragment of the larval cone-shaped lobe stained with the secondary antibody and DAPI, the primary antibody against HC1 subunit was omitted. No signal detected in the fat body cells. fb, fat body of the cone-shaped lobe; ne, nuclei of the epithelial cells covering the cone-shaped lobe; nf, nuclei of the fat body cells; nm, nuclei of the maternal fat body cells. **b**, inset, **c** paraffin sections, fluorescence microscope.

We have found no tracheal elements inside CSLs, even in the largest first instar larvae still present in the mother’s uterus. The same applies to other larval body parts (not shown). These observations in conjunction with considerable size of the *Arixenia* larvae raised a question if simple gas diffusion would be sufficient enough to provide adequate amount of oxygen to support intense larva metabolism. Therefore, we asked whether hemocyanin, the respiratory pigment occurring in some arthropod taxa, may support respiration of the *Arixenia* progeny. We first tested whether hemocyanin is present in homogenates of the first instar larvae dissected from the uterus. Immunostaining of the western blots with antibodies raised against two hemocyanin subunits (HC1 and HC2) revealed proteins with molecular weight of around 70–80 kDa (Fig. 6a). These results correspond well with the weight of the hemocyanin subunits in other arthropods (see “Discussion”). To identify tissues in which hemocyanin is expressed, we used the immunofluorescence technique. The immunostaining of paraffin sections of the CSLs and surrounding maternal tissues with antibodies against hemocyanin subunits showed a strong fluorescence signal only in cells of the fat body present in the CSL cavity (Fig. 6b, c). The analysis of the fluorescence images revealed that the hemocyanin subunits are distributed within the fat body cells in a characteristic stripy pattern (Fig. 6b). Interestingly, the maternal fat body strands attached to the uterine wall were negative (Fig. 6b, inset).

## Discussion

### The mechanism of the intrauterine respiration in *Arixenia*

Our light and electron microscopy analyses of the *Arixenia* reproductive system revealed a rich network of tracheal tubes, which penetrates the maternal tissues (the uterus) contacting the developing offspring. The ultrastructure of both tracheae and tracheoles in *Arixenia* is highly similar to these respiratory organs described in other insect species (Noirot and Noirot-Timothee 1982; Mill 1985; Chapman 1998). Comparative studies of various insect tissues indicate that the relative richness of the tracheal system is correlated with rates of oxidative metabolism in these tissues (Chapman 1998; Harrison 2003). Tracheae are particularly abundant in tissues with high aerobic metabolism, such as muscles. We believe that the elaborate tracheation of the uterine sheaths indicates demanding respiratory requirements, which are associated, at least partly, with the viviparous pattern of reproduction in *Arixenia*. We assume that, similarly to other insect systems, the bulk of oxygen transfer occurs through the thin-walled tracheoles. Thanks to thin walls, tracheoles are capable of transporting oxygen at high rates by diffusion and they are believed to be the major site for gas exchange inside the insect body (reviewed in Chapman 1998; Harrison 2003). In *Arixenia*, tracheoles insinuate themselves between muscle fibers of the ovariolar and uterine sheaths and penetrate the thick basal lamina supporting their epithelial layer (Tworzydlo et al. 2013a, 2013b, this report). Such an arrangement highly reduces the oxygen diffusion distance between tracheolar tubes and the uterine epithelium, which, in turn, remains in direct contact with the developing larvae.

It has been reported previously that the paired ovaries of *Arixenia* consist altogether of six ovarioles. In each ovariole, only one embryo develop at a given time (Tworzydlo et al. 2013a, 2013b). Because the embryonic development is always synchronized in all ovarioles, there are usually as many as six embryos/larvae developing in the uterus of a given female (Bilinski and Tworzydlo 2019; this report). These findings, combined with the large size of the first instar larvae (around 15-mm length), strongly suggest that the demand for oxygen is likely to increase considerably during the intrauterine phase of development and it cannot be easily met by a simple gas diffusion only. Our western blot assays and the immunostaining analyses demonstrated the presence of hemocyanin subunits in the *Arixenia* larvae. The presence of this respiratory protein may favor more efficient distribution of oxygen throughout a large body of the first instar larvae. We propose that the *Arixenia* hemocyanin is a bona fide oxygen-carrier protein, although further biochemical and molecular analyses will be required to fully verify this hypothesis. Based on the obtained results, we propose a biphasic mechanism of respiration in the *Arixenia* offspring, developing inside the mother’s reproductive system. In the first phase, air/oxygen is supplied to the region of the mother/larva interface by the extensive maternal tracheal system associated with the uterus wall. Subsequently, oxygen diffuses through the thin tracheole walls, passes the relatively thin CSL tissue and diffuses into the hemocyanin-enriched hemolymph of the late larvae. In the second phase, oxygen bound to hemocyanin is distributed throughout the offspring body with circulating hemolymph. However, it is not clear how well-developed the larval tracheal system is at the moment of birth, and how long after birth the *Arixenia* larvae continue to use the respiratory proteins, if at all.

It has been recently suggested that, in *Arixenia*, the intimate contact points between the uterus epithelium and lobes of the larval abdominal outgrowths constitute collectively a dispersed placental analogue (Bilinski and Tworzydlo 2019). CSLs, characterized in this report, appear to be key elements of this placental analogue by mediating and participating in the biphasic respiration of the *Arixenia* first instar larvae, as they develop within the mother’s reproductive system.

We have found that the larval fat body also contains cells with the characteristics of the urocytes or urate cells: their cytoplasm consists of large vacuoles containing crystalloid spherules, few mitochondria, and sparse ER cisternae (for a review, see Dean et al. 1985; Cohen 2003). We have not investigated the chemical composition of the spherules in the *Arixenia* urocytes; however, morphologically similar structures reported in cockroaches and certain other insects consistently contain uric acid, the insect end product of nitrogenous metabolism (reviewed in Dean et al. 1985; Chapman 1998). We assume therefore that the spherules, present in the *Arixenia* urocytes, may serve as depots of uric acid sequestrated either for subsequent use of nitrogen or elimination from the body. In the latter case, urocytes could be engaged in excretion, in conjunction with the Malpighian tubules (for a thorough discussion of excretion in *Arixenia* larvae, see Jaglarz et al. 2018).

### Hemocyanin and respiration in insects

Hemocyanins are large copper-containing proteins participating in transport of oxygen in hemolymph or blood in mollusks and some arthropods (reviewed in Markl and Decker 1992, van Holde and Miller 1995; van Holde et al. 2001; Burmester 2002). Hemocyanins occur as hexamers or multi-hexamers built of six similar subunits, each of which has molecular weight around 70–80 kDa and binds one oxygen molecule (reviewed in van Holde and Miller 1995; Burmester 2002). The exact site of hemocyanin synthesis has been established only in a few species, but in all studied cases the process occurs in mesenchymally derived cells (reviewed in Markl and Decker 1992). At least in the scorpion *Androctonus* and the spiny lobster *Panulirus*, rough ER is implicated in hemocyanin synthesis and/or modification (Markl and Decker 1992). The same results were reported for the synthesis of hemocyanin in the slug *Limax* sp. (Reger 1973). Our ultrastructural analysis revealed that adipocytes of the larval fat body cells contain a network of highly distended cisternae of the rough ER filled with granular material. These features indicate high activity of ER network in synthesis of proteins destined for export from the cell and our results suggest that hemocyanin may be a good candidate for such a protein. The immunofluorescence stainings with anti-hemocyanin antibodies demonstrated the presence of this protein in the fat body residing inside the cavity of CSLs. The staining pattern of the fat body cells correlates with the distribution of the rough ER revealed by ultrastructural analysis. The abundant glycogen aggregates may be used, among others, as an energy source required for the synthesis of hemocyanin subunits and their assembly into larger complexes. It is worth noting here, that in *Arixenia*, mother’s fat body tissue does not label with anti-hemocyanin antibodies, and that the ultrastructure of adipocytes is markedly different from the larval fat body cells. In conclusion, our data lead to a suggestion that the larval fat body tissue, present inside CSLs, is the site of synthesis of hemocyanin subunits, which are subsequently transferred into the larval hemolymph.

### Phylogenetic considerations

It is well established that among arthropods, the hemocyanins are widespread in chelicerates and malacostracan crustaceans; however, this respiratory pigment has been also discovered in certain species of Onychophora, Myriapoda, and Hexapoda (reviewed in Burmester 2002). Molecular phylogenetic analyses indicated a close relationship between hemocyanins found in Hexapoda and Crustacea, further supporting the Pancrustacea taxon and suggesting a common origin of the arthropod hemocyanin family of proteins (Beintema et al. 1994; Burmester 2001). In a recent comprehensive survey of hexapod orders, the presence of hemocyanin mRNA has been confirmed in representatives of most ametabolous and hemimetabolous hexapods, including Dermaptera (i.e., *Chelidurella acanthopygia*) (Pick et al. 2009). Here, we report the hemocyanin occurrence in *A. easu*, which indicates that hemocyanin might be ubiquitous in dermapterans.

The role hemocyanin plays in insects with the tracheal respiratory system remains mostly a mystery. Recently, however, functional hemocyanin has been identified in the hemolymph of the stonefly *Perla marginata* (Plecoptera) and the firebrat *Thermobia domestica* (Zygentoma) (Hagner-Holler et al. 2004; Fochetti et al. 2006; Pick et al. 2008). The presence of hemocyanin in plecopterans was attributed to the semiaquatic lifecycle of these insects (Hagner-Holler et al. 2004). As *A*. *esau* is an exclusively terrestrial insect, we suggest that the presence of hemocyanin in this species is related to the viviparous type of reproduction. Furthermore, we speculate that during gradual evolvement of the matrotrophy in *Arixenia*, the presence of the ancestral hemocyanin had been exploited to facilitate respiration of larvae developing in the environment deprived of adequate oxygen supply, i.e., inside the mother’s reproductive system. The use of the ancestral hemocyanin may have had a great adaptive value by creating favorable conditions for enhanced metabolism and increasing the survivorship during larval development inside mother’s body.

## Conclusions

Our results demonstrate that respiration in *Arixenia* larvae relies on a biphasic mechanism, which involves both the maternal tracheal system and a respiratory pigment (hemocyanin) present in the body cavity of offspring. Characteristic CSLs of the larval abdominal outgrowths participate in the formation of a placenta-like organ, which mediates gas exchange between the maternal and larval organisms. Interestingly, *Arixenia* adaptations to physiological challenges of matrotrophic viviparity are realized differently with respect to respiration and excretion. In the latter case, the larvae appear completely independent of the maternal organism in managing metabolic waste removal (Jaglarz et al. 2018). This indicates a highly plastic response of *Arixenia* to distinct challenges associated with the viviparous mode of reproduction.
